# Detection of *PIK3CA* Gene Mutation in Head and Neck Squamous Cell Carcinoma Using Droplet Digital PCR and RT-qPCR

**DOI:** 10.3390/biom11060818

**Published:** 2021-05-31

**Authors:** Edyta M. Borkowska, Magda Barańska, Magdalena Kowalczyk, Wioletta Pietruszewska

**Affiliations:** 1 Department of Clinical Genetics Chair of Laboratory and Clinical Genetics, Medical University of Lodz, 92-213 Lodz, Poland; edyta.borkowska@umed.lodz.pl; 2 Department of Otolaryngology, Head and Neck Oncology, Medical University of Lodz, 93-143 Lodz, Poland; magda.a.baranska@gmail.com (M.B.); magdalena.marianna.kowalczyk@gmail.com (M.K.)

**Keywords:** head and neck squamous cell carcinoma, *PIK3CA* gene, human papilloma virus, *BRCA*, p16

## Abstract

Head and neck squamous cell carcinomas (HNSCC) are the seventh cause of human malignancy with low survival rate due to late diagnosis and treatment. Its etiology is diverse; however genetic factors are significant. The most common mutations in HNSCC were found in the genes: *PIK3CA* (10–12%), *BRCA1* (6%), and *BRCA2* (7–9%). In some cases, these biomarkers correlate with recurrences or survival showing a potential of prognostic and predictive value. A total of 113 formalin-fixed paraffin embedded (FFPE) tumor samples were collected from patients with HNSCC (oral cavity: 35 (31.0%); oropharynx: 30 (26.0%); larynx: 48 (43.0%)). We examined *PIK3CA* H1047R mutation by Real Time PCR (RT-qPCR) and droplet digital PCR (ddPCR). *BRCA1* and *BRCA2* mutations were analyzed by RT-qPCR while p16 protein expression was assessed by immunohistochemistry. Finally, we identified HPV infection by RT-qPCR. The relationships between genomic alterations and clinical parameters were assessed using the Yates’ corrected Chi-squared test or Fisher’s exact test for nominal variables. Kaplan Meier plots were applied for survival analysis. Our results revealed 9 *PIK3CA* H1047R mutations detected by ddPCR: 8 of them were negative in RT-qPCR. Due to the use of different methods to test the presence of the *PIK3CA* gene mutation, different treatment decisions might be made. That is why it is so important to use the most sensitive methods available. We confirmed the usefulness of ddPCR in the *PIK3CA* mutation assessment in FFPE samples.

## 1. Introduction

Cancer detection, treatment and prevention was, until recently, something of a “one-size-fits-all”. Current practice guidelines recommended chemotherapy based on risk and not on predictive factors. The genetic signature of cancer may help predict the risk of recurrence or identify the best treatment strategy. The number of gene mutations implicated in cancer is growing and the number of drugs being developed to target specific mutations is also rising. Modern technologies offer personalized cancer therapies [1]. However, they are not fully available to patients with head and neck squamous cell carcinomas (HNSCC)—a group of malignant tumors of that region, excluding the brain and eyeball. HNSCC accounts for 5% of all cancer cases and is the seventh cause of human malignancy [2]. Worldwide, the number of patients who are diagnosed with HNSCC is estimated to be 800,000, with 430,000 deaths every year [2,3]. More than 90% of head and neck cancers originate from squamous epithelial cells, and affect mostly the upper respiratory tract, including larynx (24%), oral cavity (40%) and pharynx (35%). They are most often recognized and treated at an advanced stage. Despite advances in diagnostic and therapeutic techniques, the 5-year survival rate has not improved for decades and remains low [4,5,6].

Etiology is multifactorial, both the genetic and environmental factors participate in the tumorigenesis, with smoking and alcohol consumption being the main causative factors of head and neck cancer. HPV is also an important contributing factor particularly of oropharyngeal cancer and traditionally concerns younger, healthier patients with high economic status and high-risk sexual behavior. It is related to improved prognosis and deintensification treatment strategies are being evaluated in ongoing clinical trials. HPV status evaluation has been incorporated in treatment guidelines worldwide as a major prognosis impact which is also reflected on the new Tumor Node Metastasis (TNM) staging for HPV+ cancer [7].

Head and neck cancers, as multistep genetic diseases, require several genetic alterations to transform from precancerous lesion to invasive carcinoma [8]. According to the literature, the phosphoinositide 3-kinase (PI3K) oncogenic pathway is the most commonly mutated in HNSCC (46/151—30.5%) and can be an attractive therapeutic target since there are several inhibitors of PI3K and other mediators in clinical use or undergoing clinical trials [9]. PIK3CA inhibitors, therefore, are a promising drug class that may provide treatment success. Multiple studies might indicate that mutations of *TP53*, *CDKN2A*, *PTEN*, *BRCA* and *PIK3CA*, could act as a “driver changes” in HNSCC. The last one, which encodes p110α-the catalytic subunit of phosphatidylinositol 3-kinase (*PIK3CA*), is an important regulator of cellular growth, proliferation, metabolism, migration and apoptosis. [10,11]. *PIK3CA* is among the most frequently mutated genes in HNSCC, affected both in HPV-positive and negative disease (56 and 34% respectively) [12,13]. Over 70% of its mutations are located in three hotspots: E542, E545 in the helical domain and in H1047 in the kinase domain [14,15,16,17]. Moreover, *BRCA1* and *BRCA2* are among top 30 altered genes in HPV-positive and *BRCA 2* in HPV-negative HNSCC [18]. *BRCA1* mutations are seen in 6%, and BRCA2 in 7–9% of HNSCC (7.8% in HPV-positive tumors). Additionally, *BRCA1/2* mutations appeared almost exclusively as co-mutations with *PIK3CA* or *TP53* [19,20]. The frequency of *PIK3CA* mutations in head and neck cancer was identified in the literature in 10% of patients [21,22].

Except for the evaluation of HPV viral DNA integration into the host cell, p16 protein accumulation in the proliferating cell layers is an alternative marker for HPV infection.

Tumor staging for HNSCC based on molecular signatures is not commonly used in treatment decision making, although it can be very informative from a biological perspective [19,23,24]. Different methodologies have proven their usefulness for the evaluation of archival tumor tissues, where poor DNA quality and limited sample availability are major obstacles. The reasons for discordance between different techniques are not clear and are difficult to delineate definitively. In this study, we conducted a performance comparison of two highly sensitive molecular techniques for detecting *PIK3CA* mutation: real time quantitative polymerase chain reaction (RT-qPCR) and droplet digital PCR (ddPCR) which represents an enrichment strategy that allows for the detection of low-level mutations by amplification of single DNA molecules. The greatest advantage of ddPCR is that it does not depend on the cycle threshold of the amplification curve, therefore it is not affected by errors related to the multiplication of DNA. Requiring low amounts of DNA input, ddPCR has unique features that make it superior to RT-qPCR. ddPCR is faster and shows less technical variability between experiments, providing better, more accurate, reproducible and less ambiguous results in comparison to standard RT-qPCR [25]. Molecular markers determined in tumor samples provided information for the detection, monitoring or response to treatment in a group of patients [26,27]. Mellert H et al found that a ddPCR-based genomic test was able to evaluate 94% of samples within 72 h [28]. They demonstrated clinical validation results for *EGFR* mutations (exon 19 deletions, L858R and T790M), *KRAS* mutations (G12C, G12D, G12V), and *EML4-ALK* fusion with sensitivity and specificity of 90.9 percent and 100 percent, respectively. Results for liquid biopsy was 97.0 percent concordant with tissue. The usefulness of RT-qPCR and ddPCR methods has been confirmed for the detection of pathogens, as well as for the detection of germinal and somatic mutations in numerous neoplasms [29,30,31,32,33]. The aim of that study was to evaluate the usefulness of selected methods in the assessment of the mutation H1047R of *PIK3CA* gene. We also assessed the most frequent mutations of *BRCA1/2*, infection of HPV virus and p16 expression in patients’ samples to get a picture of the biological potential of the tumor.

## 2. Materials and Methods

### 2.1. Group Characteristics

Tumor specimens were collected from 113 patients diagnosed with head and neck squamous cell carcinoma surgically treated at the Department of Otolaryngology, Head and Neck Oncology, Medical University of Lodz, from 2010 to 2016. The group consisted of Caucasians from the same residential area. Patients were staged using TNM staging system in accordance with the 7th edition and subsequently re-evaluated in accordance with the 8th edition of the American Joint Committee on Cancer Staging of Head and Neck Cancer [7,34,35]. There was survival data available for all of the group. Patients whose death was not related to the primary disease were excluded from the analysis. The observation time varied from 2 to 167 months with a mean of 54.9 months. Time from primary surgical treatment to the onset of local or nodal recurrences was found in a total of 13 patients (11.60%) over a period from 6 months to 9 years (mean 40.9 months; SD 25.13; median 24 months). Patterns for age, sex, primary tumor location, clinical staging, grading, local and nodal recurrence were correlated with HPV status. Tobacco exposure was expressed as pack-years of smoking. We distinguished between nonsmokers and smokers, among whom we differentiated light and heavy, respectively, below and above the median (7300 cigarettes per year), due to the same value in all groups. Patients due to alcohol consumption were grouped into non-drinkers and those who drank less or more than 6 beverages weekly (median). The demographic and clinical data are described in Table 1. The study was approved by the Local Ethics Committee (RNN/12/06/KE; RNN/69/07/KE). All procedures performed in studies involving human participants were under the ethical standards of the institutional and national research committee and in agreement with the 1964 Helsinki Declaration and its later amendments or comparable ethical standards. Informed consent was obtained from all individual participants involved in the study.

### 2.2. DNA Isolation

Formalin-fixed paraffin embedded (FFPE) tumor samples were obtained from HNSCC tumors collected between 2010–2016. Written consent was obtained from all of the patients. All tumor samples were assessed by sectioning, hematoxylin and eosin staining under a microscope to ensure that the tumor content in each sample was higher than 50%. DNA was isolated from samples using Maxwell RSC DNA FFPE Kit (Promega Corporations, Madison, WI, USA, cat No AS1450) and automatic isolation on AS4500 device (Promega Corporations, Madison, WI, USA). The purity of extracted genomic DNA was checked by measuring the absorbance at 260 nm(A_260_), 280 nm (A_280_) and 230 nm (A_230_) with a Nanodrop 2000 spectrophotometer. Extracted genomic DNA with a A_260_/A_280_ ratio between 1.8–2.0 and A_260_/A_230_ ratio over 2.0 were considered satisfactory to conduct analysis.

### 2.3. p16 Immunohistochemical Expression

The CINtec Histology Kit (Ventana Medical Systems, Oro Valley, AZ, USA, Roche) was used for p16 immunohistochemistry, following manufacturer’s protocol. The evaluation was performed according to the previously published criteria [36]. The sample was considered positive when greater than 70% of carcinoma tissue was detected with strong and diffuse nuclear and cytoplasmic immunostaining for p16. Lack of reactivity or faintly diffuse was counted as p16-negative. Independent assessment by two investigators was used for staining intensity and the percentage of positively stained cells for each analyzed slide.

### 2.4. Genotyping of the HPV

Real Time Kit V12-100 FRT CE IVD (Sacace Biotechnologies, Milan, Italy) was used for quantitative detection and genotyping of Human Papillomavirus (genotypes 16 and 18). Briefly, PCR reactions were carried out in a volume of 25 µL, using 10 µL of isolated DNA (5 ng/µL), 7.0 µL of PCR-mix-1-FRT and 8.0 µL of Mix (PCR-mix-2 buffer and Taq DNA Polymerase). The thermal cycling conditions were 95 °C for 15 min followed by 40 cycles at 95 °C for 5 s, 60 °C for 30 s and 72 °C for 15 s. Internal Control (Human DNA–β-globine) was detected on the HEX channel, HPV 16 on the FAM channel and HPV 18 on ROX channel.

### 2.5. Quantitative PCR (RT-qPCR) for PIK3CA and BRCA1/2 Mutations Assessment

The RT-qPCR for the amplification of exon 20 of *PIK3CA* gene and detection the mutation H1047R was performed using 25 ng DNA in total volume of 25 µL as previously described [37]. Briefly, the reaction includes 12.5 µL TaqMan Universal Master Mix (Thermo Fisher Scientific, Waltham, MA, USA, cat no A30872), 2.5 µL 10× primers/probes mix (Thermo Fisher Scientific, Waltham, MA, USA, Cat no 4351379), 5 µL nuclease free water and 5 µL DNA 5 ng/µL. The PCR protocol was an initial enzyme activation step of 2 min at 5 °C and 10 min at 95 °C for denaturation, followed by 50 cycles of 95 °C for 15 s and 60 °C for 60 s. All reactions were performed in duplicates using the Real Time PCR System CFX 96 (BioRad Laboratories Inc., Hercules, CA, USA). Mutations of genes *BRCA1/2* were detected using Oncogenetic BRCA Panel (Sacace Biotechnologies, Milan, Italy, cat no R-27/P-48FRT) according to manufacturer’s protocol. The panel includes mutations: gene *BRCA1* (185delAG, 4153delA, 5382insC, 3819delGTAAA, 3875delGTCT, T181G (Cys61Gly), 2080delA) and gene *BRCA2* (6174delT). Briefly, it is a quantitative test that allows the detection by RT-qPCR based on the amplification of the genome-specific region using specific primers. In each reaction mutant (FAM probe) and wild type (HEX probe) of the allele is assessed. The PCR reactions were run in the following temperature program: 80 °C for 2 min, 94 °C for 3 min followed by 40 cycles of 95 °C 10 s and 60 °C for 40 s.

### 2.6. Droplet Digital PCR (ddPCR)

Analysis by ddPCR was performed on the QX100 Droplet Digital System (BioRad Laboratories Inc., Hercules, CA, USA), including primers and probes (FAM-mutant type BioRad cat no 10031246 and HEX-wild type BioRad cat no 10031249) and Supermix for probes (No dUTP) according to manufacturer’s protocol [38]. Briefly, reactions were performed in 25 µL volume using ddPCR 2× Master mix (BioRad Laboratories Inc., Hercules, CA, USA), 1.25 µL 20× primer and probe mix, 8.75 µL nuclease free water and 2.5 µL template. Then 20 µL of each sample was transferred into the middle well of the cartridge (BioRad cat no 1864008) and 70 µL of droplet generation oil (BioRad cat no 1863005) was added to the lower well. Once the process of generating droplets was completed, 40 µL of mixture was transferred into the wells of a 96-well plate, sealed (BioRad cat no 1814040) and loaded in a thermal cycler (T100-BioRad Laboratories Inc., Hercules, CA, USA). PCR was performed using following conditions: 95 °C for 10 min, followed by 40 cycles of 94 °C for 30 s and 57 °C for 60 s. After thermal cycling the plate was read in the QX200 Droplet Reader and based on positive droplets and the Poisson distribution, the absolute copy number of the mutant allele of *PIK3CA* H1047R and wild type was calculated (QuantaSoft analysis system BioRad Laboratories Inc., Hercules, CA, USA).

### 2.7. Statistical Methods

Relationship between genomic alterations and clinical parameters were assessed by using the Yates’ corrected Chi-squared test or Fisher’s exact test for nominal variables. (STATISTICA 13, Stat-Soft Inc.). Differences were considered significant at confidence intervals greater than 95% (*p* < 0.05). Survival distribution were estimated by the Kaplan−Meier method. Overall survival (OS) was determined from the time of initial diagnosis to the time of death whereas recurrence-free survival (RFS) as time from the initial diagnosis to recurrence. The statistical significance of differences between survival rates was ascertained using the log-rank test. Univariate Cox proportional hazard models were performed to assess the prognostic value of clinical and genomic markers on OS. The results are presented as hazard ratio and 95% confidence interval.

## 3. Results

The 113 cases represent primary tumors located in the oral cavity (n = 35; 31.0%), the oropharynx (n = 30; 26.0%) and in the larynx (n = 48; 43.0%). With regard to the patients’ data, male to female ratio was 76% versus 24%, respectively, with the average age of the patients 63.4 (ranged 42−91). This represents the normal distribution in that disease. In terms of habits, 74% were smokers or former smokers and 49% had a history of alcohol consumption. This cohort included comparable numbers of tumor stage T1−T2 (52%) and T3−T4 (48%) tumors. The prevalence of patients without metastases to the neck lymph nodes (70%) was observed.

**Figure 1:**
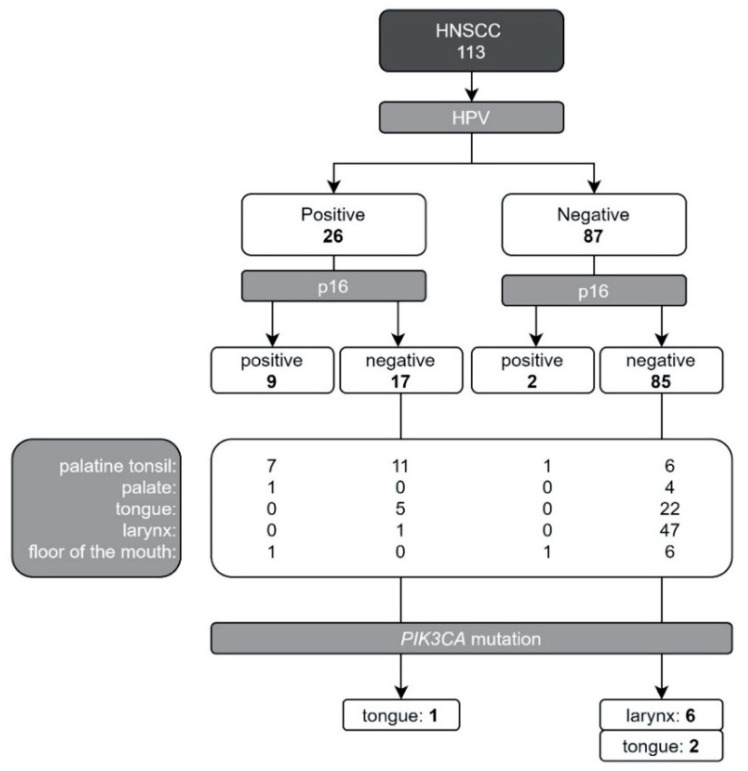
Study group constitution considering tumor primary location and genetic testing results (*PIK3CA* H1047R mutation, HPV infection and p16 protein expression; Abbreviations: HNSCC—head and neck squamous cell carcinoma, HPV—human papilloma virus, *PIK3CA*-phosphatidylinositol-4,5-bisphosphate 3-kinase catalytic subunit alpha gene).

All patients were pathologically diagnosed, and the collected tissues were tested for HPV (26/113 HPV-positive); p16 protein (11/113 abnormal expression); *PIK3CA* mutation (9/113—8%) and *BRCA1/2* mutation (0/113). Data concerning HPV infection and p16 expression in correlation with *PIK3CA* mutation is shown in Figure 1. Most of the observed *PIK3CA* mutations (8/9, 88.9%) occurred in patients without confirmed HPV infection (8/87, 9.2% in the HPV negative group) and with normal p16 expression (N = 85) in patients with laryngeal cancer. In the HPV positive group there was one patient with *PIK3CA* mutations (1/26, 3.85%), diagnosed with tongue cancer and abnormal expression of p16 protein (Figure 1).

Analyzing the variables presented in Table 2, we obtained significant differences only for the correlation with the clinical stage of the disease, the stage of histopathological grade and metastases. We observed a two-fold higher risk of neck metastases in the group of women compared to men. At the same time, people with neck nodal involvement had over thirteen times higher risk of death. Patients with advanced grade (G3) of the disease had more than four times higher risk of neck metastasis. However, HPV-negative patients had more than three times lower chance of neck metastases. This is interesting because usually a better prognosis is observed in the group of people who develop the disease based on HPV infection. Moreover, patients with normal expression of the p16 protein in tumor tissue had a more than five times higher chance for a better disease course (stage T1−T2). 

**Figure 2:**
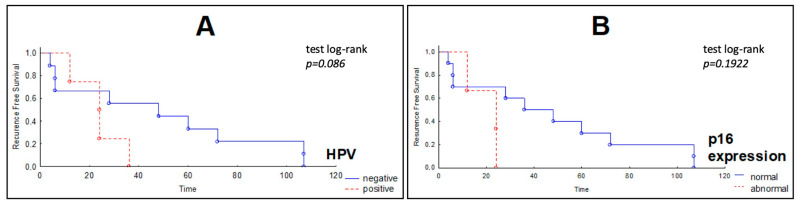
Kaplan−Meier curves of recurrence-free survival (RFS) of HNSCC patients according to (**A**) HPV infections and (**B**) p16 expression. Patients with p16 abnormal expression in tumors show a worse outcome (the *X* axis—the survival time in months, *Y* axis—the RFS).

Univariate Cox regression analysis presented in Table 3 did not show any significant correlations between the analyzed variables. Kaplan−Meier survival curves for recurrence free survival (RFS) in the group of patients with HNSCC showed no correlation between the studied variables (*PIK3CA* mutation, HPV infection and p16 expression). However, in the group of patients with the normal expression of the p16, we observed a better outcome (log-rank test *p* = 0.086) (Figure 2).

Comparison between RT-qPCR and ddPCR shows that for only one case did both methods give equivalent results. Eight ddPCR-positive cases were negative at RT-qPCR. We did not observe any correlation for the presence of the *PIK3CA* H1047R gene mutation and tested variables. 

## 4. Discussion

Current evidence suggests that *PIK3CA* mutations have predictive and prognostic value, although their clinical significance remains unclear [14]. The mutational spectrum that has been reported in the literature by tissue tumor sequencing, with *TP53* and genomic alterations in the PI3K pathway being among the most frequent events in HNSCC [39]. In our study, we compared two sensitive molecular methods and we found that ddPCR detected mutations with higher sensitivity in FFPE samples from HNSCC patients (RT-qPCR detected mutation in one case and ddPCR in nine cases). A total of nine mutations were detected in the patients’ group, which is in line with the frequency of these changes reported for head and neck cancers (8%). The majority of them (8/9, 88.9%) were observed in HPV negative cases of HNSCC. *PIK3CA* mutations in the helical domain (E542K, E545K) were more often observed in HPV positive samples of HNSCC while in HNSCC HPV negative cases, *PIK3CA* mutations were mostly found across the entire gene. [22]. We confirmed the H1047R mutation (located in kinase domain) in exon 20 of *PIK3CA* gene mainly in HPV negative HNSCC (only 1/9 in HPV positive HNSCC). Nichols et al. found three H1047R mutations in 87 analyzed patients, which is 3.45% (all three mutations were detected in HPV negative cases) [22]. In line with our results other studies have reported a low concordance rate for these two methods [40,41,42]. Lui et al reported 12.6% mutations of *PIK3CA* in HNSCC (46/151), which was substantially fewer than the number reported by TCGA (in total 21%, 58/279 cases, including: 32/279, 11.7% of E545K/E542K mutations and 26/279, 9.3% of H4047R mutation) [9]. Our result of 8% for the H1047R mutation in the *PIK3CA* gene remains consistent with the data from the TCGA database [9,12]. Beavers et al. reported that a point mutation (*PIK3CA* E545K), detected at a fractional abundance of 28.9% in the primary tumor tissue by ddPCR, could not be identified by Sanger sequencing. Due to the use of different methods to test for the presence of the *PIK3CA* gene mutation and the failure to detect an existing mutation, the wrong patient treatment decision might be made. These authors suggested that when FFPE DNA was used for PCR and Sanger sequencing, a phenomenon of “allelic drop out” could occur, due to sample degradation. ddPCR was less prone to this effect because of the smaller amplicon size used in this technique. The ddPCR was also a reliable, highly sensitive alternative method for the detection of the *BRAF*^V600E^ mutation in papillary thyroid carcinoma (it confirmed the presence of the mutation in 92 samples whereas Sanger sequencing in 67 samples) [43]. The authors concluded that Sanger sequencing is a gold standard and a widely used method in laboratories for detecting mutations but because of its relatively low sensitivity (in 25 samples Sanger sequencing indicated wild-type *BRAF* instead of mutated) the detection requires a large amount of tumor DNA in the sample [43]. Wang et al. compared the results from Sanger direct sequencing (as the standard), ddPCR, and quantitative real-time PCR (qRT-PCR) [43]. Their data indicated that ddPCR was much more sensitive and much easier to interpret than qRT-PCR [44]. The results published by Zhang et al. demonstrated that the ddPCR approach reliably detected plasmid samples with 5% (398 copies), 1% (57 copies), 0.5% (24 copies) and even 0.1% (average 6 copies) mutation rate whereas qPCR at the levels of 5 and 1% [45]. The other results revealed that both methods had high analytical sensitivity and they were capable of detecting the *JAK2* V617F mutation with a limit of detection of 0.12% for RT-qPCR and 0.01% for ddPCR [46]. The latter work showed the significant differences in the possibilities of detecting mutations of both techniques. This is in line with the results we obtained. The differences between methods are presented in Table 4. They show that for HNSCC and breast cancer, the *PIK3CA* gene mutation detection tests were conducted in various biological materials (FFPE, serum, plasma, frozen tissue, cell lines) and with the use of various research techniques (RT-qPCR, Sanger sequencing, NGS, ddPCR) which affected the received sensitivity of detected changes [19,20,23,47,48,49,50,51,52,53,54,55,56]. This translates into the quality and quantity of the obtained results (including false negative and false positive). At the moment the Sanger sequencing has been the gold standard for verifying such mistakes. Unfortunately, this is no longer possible due to the aforementioned limitations of this method. Patients waiting for treatment need a selected type of therapy, based on the molecular profile of the tumor. Techniques such as ddPCR and NGS make it possible more and more often, although of these two methods the latter is certainly cheaper [57,58,59].

In our group of patients, we did not detect any of the eight *BRCA1/2* mutations tested. Comparable results were obtained by Moslehi et al. in a study of Ashkenazy Jews. In the group of patients with HNSCC, they found an exceptionally low (0%) frequency of *BRCA1/2* germline mutations despite the fact that in this population there is a founder effect for these changes [60]. In the Polish population we also have such an effect described. Perhaps mutations inversely correlate with cancers of the neck and head. In terms of the characteristics of the study group, with regard to HPV and p16 protein expression, our results were consistent with those described earlier. Only the correlation of the absence of HPV infection with a better prognosis is an interesting observation. Taking into account the survival curves, we did not find any correlation with the analyzed variables either, except for the trend related to better prognosis in patients with normal expression of the p16 protein. In Garcia-Esudero et al. studies the presence of mutation or amplification of *PIK3CA* gene was also not associated with an increased risk of recurrence [61].

It is necessary to mention some limitations of our study. First of them is that we did not confirm the presence of mutations in samples by sequencing. The reason for this was the fact that in recent studies, ddPCR was found to be more precise than sequencing for detecting rare alleles as molecular markers. Sequencing strategies applied to FFPE samples for mutation detection pose serious problems derived from polymerase error rates. This enzyme introduces mistakes during amplification, generating point mutations. An estimated error rate of 1% gives rise to hundreds of millions of sequencing mistakes in a single experiment [62]. As a result, at present in comparison of mutation detection in the clinical setting there is no gold standard available for assessment of tumor heterogeneity. Furthermore, we assessed only one type of mutations of *PIK3CA* and we did not check expression on mRNA levels which would be extremely valuable, and we intend to do so because Garcia-Escudero et al. revealed that *PIK3CA* overexpression, but not mutations, is a poor prognostic marker in HNSCC. There is a need for a larger study to validate our results [61].

## 5. Conclusions

We confirmed the utility of ddPCR for detection of *PIK3CA* mutation in FFPE samples. Differences in somatic events between individuals as well as within the same single biopsy site, and the method of detection contribute to treatment failure and drug resistance because of the vast genetic variety between samples. The ddPCR technique appears to be a more sensitive than RT-qPCR and should be routinely implemented to determine the presence of genetic markers because it prevents false negative results.

## Figures and Tables

**Table 1:** Study group characteristics (Abbreviations: HNSCC—head and neck squamous cell carcinoma, HPV—human papilloma virus).

Patient Characteristics	All HNSCC (n = 113)	HPV-Positive (n = 26)	HPV-Negative (n = 87)
Age, y			
Average (range)	63.4 (42–91)	61.6 (42–83)	64.0 (42–91)
Sex (%)			
Male	86 (76)	17 (63)	70 (80)
Female	27 (24)	10 (37)	17 (20)
Tobacco, no. (%)			
none	29 (26)	9 (35)	20 (23)
light	19 (17)	4 (15)	15 (17)
heavy	65 (57)	13 (50)	52 (60)
Alcohol, no. (%)			
none	58 (51)	15 (58)	43 (49)
light	45 (40)	10 (38)	35 (40)
heavy	10 (9)	1 (4)	9 (11)
Tumor sites in head and neck, no. (%)			
Palatine tonsil	25 (22)	18 (69)	7 (8)
Tongue	27 (24)	5 (19)	22 (25)
Palate/Pharynx	5 (4)	1 (4)	4 (5)
Larynx	48 (43)	1 (4)	47 (54)
Floor of mouth	8 (7)	1 (4)	7 (8)
Tumor stage, no. (%)			
T1–T2	59 (52)	13 (50)	46 (53)
T3–T4	54 (48)	13 (50)	41 (47)
Nodal stage, no. (%)			
N0	80 (70)	13 (50)	67 (77)
N+	33 (30)	13 (50)	20 (23)
TNM, no. (%)			
I–II	55 (49)	12 (46)	43 (49)
III–IV	58 (51)	14 (54)	44 (51)
Grading, no. (%)			
G1	13 (12)	4 (15)	9 (10)
G2–G3	100 (88)	22 (85)	78 (90)
Recurrence, no. (%)	13 (11)	4 (15)	9 (10)
p16, no. (%)	11 (10)	9 (35)	2 (2)

**Table 2:** Characteristics of the patient group and the impact of the clinical stage of the disease, the presence of metastases, HPV infection and p16 protein expression on selected variables. (*p* < 0.05 was bolded; abbreviations: OS—overall survival; HPV—human papilloma virus).

			T1–T2 vs. T3–T4			Nodal Stage N0 vs. N+			HPV Negative (−) vs. HPV Positive (+)			p16 Normal vs. Abnormal Expression		
		All (n = 113)	T1–T2 (n = 57)	T3–T4 (n = 56)	*p* Value	N0 (n = 80)	N+ (n = 33)	*p* Value	HPV− (n = 87)	HPV+ (n = 26)	*p* Value	p16 = 0 (n = 102)	p16 = 1 (n = 11)	*p* Value
Sex														
	Male	86 (76.0%)	42 (73.7%)	44 (78.6%)	0.542	65 (81.3%)	21 (63.6%)	**0.047**	70 (80.5%)	16 (61.5%)	0.084	79 (77.5%)	7 (63.3%)	0.5165
	Female	27 (24.0%)	15 (26.3%)	12 (21.4%)		15 (18.8%)	12 (36.4%)		17 (19.5%)	10 (38.5%)		23 (22.6%)	4 (36.4%)	
Survival OS														
	alive	84 (74.3%)	49 (86.0%)	35 (62.5%)	**0.006**	62 (77.5%)	11 (39.4%)	**0.000**	66 (75.9%)	18 (69.2%)	0.7606	78 (76.5%)	6 (54.6%)	0.6278
	death	29 (25.7%)	8 (14.0%)	21 (37.5%)		9 (22.5%)	20 (60.6%)		21 (24.1%)	8 (30.8%)		24 (23.5%)	5 (45.5%)	
Recurrence														
	Yes	13 (11.5%)	10 (17.5%)	3 (5.3%)	**0.042**	10 (12.5%)	3 (9.1%)	0.847	9 (10.3%)	4 (15.4%)	0.7215	10 (9.8%)	3 (17.3%)	0.2195
	No	100 (88.5%)	47 (82.5%)	53 (94.7%)		70 (87.5%)	30 (90.9%)		78 (89.7%)	22 (84.6%)		92 (90.2%)	8 (81.8%)	
HPV														
	negative	87 (77.0%)	45 (79.0%)	42 (75.0%)	0.618	67 (83.8%)	20 (60.6%)	**0.008**				85 (83.3%)	2 (8.2%)	**0.0000**
	positive	26 (23.0%)	12 (11.0%)	14 (25.0%)		13 (16.3%)	13 (39.4%)					17 (16.7%)	9 (81.8%)	
Smoking														
	Yes	84 (74.3%)	35 (61.4%)	49 (87.5%)	**0.001**	59 (73.8%)	25 (75.8%)	0.825	67 (77.0%)	17 (65.4%)	0.2357	76 (74.5%)	8 (72.7%)	0.8144
	No	29 (25.7%)	22 (38.6%)	7 (12.5%)		21 (26.3%)	8 (14.3%)		20 (13.0%)	9 (34.6%)		26 (25.5%)	3 (17.3%)	
Alkohol														
	Yes	55 (48.67%)	22 (38.6%)	33 (58.9%)	**0.031**	37 (46.3%)	18 (54.5%)	0.422	44 (50.7%)	11 (42.3%)	0.4573	52 (51.0%)	3 (17.3%)	0.2391
	No	58 (51.33%)	35 (61.4%)	23 (41.1%)		43 (53.8%)	15 (45.5%)		43 (49.3%)	15 (57.7%)		50 (49.0%)	8 (72.7%)	
Grade														
	G1	15 (13.3%)	10 (24.1%)	3 (5.3%)	**0.036**	12 (15.0%)	3 (9.1%)	0.161	10 (11.5%)	5 (19.2%)	0.686	11 (12.8%)	2 (8.2%)	0.8343
	G2G3	98 (86.7%)	45 (76.0%)	53 (94.7%)		68 (85.0%)	30 (90.9%)		77 (88.5%)	21 (80.8%)		89 (87.3%)	9 (81.8%)	
Tumor														
	T1T2					55 (68.8%)	2 (6.1%)	**0.000**	45 (51.7%)	12 (46.2%)	0.6181	55 (53.9%)	2 (8.2%)	**0.0249**
	T3T4					25 (31.3%)	31 (93.3%)		42 (48.3%)	14 (53.8%)		47 (46.1%)	9 (81.8%)	
Nodal stage														
	N+	33 (29.2%)	2 (3.5%)	31 (55.4%)	**0.000**				20 (13.0%)	13 (50.0%)	**0.0081**	25 (24.5%)	8 (72.7%)	**0.027**
	N−	80 (70.8%)	55 (96.5%)	25 (44.6%)					67 (77.0%)	13 (50.0%)		77 (75.5%)	3 (17.3%)	

**Table 3:** Univariate Cox regression analysis of potential predictor variables (*p* < 0.05 was bolded; abbreviations: HR—hazard ratio, CI—confidence intervals, HPV—human papilloma virus).

	Time to Survival in All Patients (n = 97)			Time to Recurrence in All Patients (n *=* 113)		
	Beta	HR (95%CL)	*p*-Value	Beta	HR (95%CL)	*p*-Value
Gender	0.288	1.33 (0.56–3.18)	0.514	1.778	5.92 (0.82–42.65)	0.077
Age	−0.013	0.98 (0.94–1.03)	0.54	0.045	1.046 (0.94–1.16)	0.391
Tumor (T1T2–T3T4)	−1.136	0.32 (0.14–0.73)	**0.007**	−1.126	0.32 (0.06–1.61)	0.169
Grade (G1–G2G3)	−0.666	0.51 (0.12–2.21)	0.371	−0.409	0.66 (0.14–3.13)	0.605
Grade (G1G2–G3)	0.994	2.70 (0.92–7.88)	0.068	1.177	3.24 (0.58–17.91)	0.177
Smoking	0.38	1.46 (0.50–4.23)	0.482	−0.178	0.83 (0.17–4.01)	0.823
Alcohol	0.551	1.73 (0.80–3.75)	0.161	−0,853	0.42 (0.13–1.42)	0.165
Chemiotherapy	2.721	15.18 (6.33–36.37)	**0.00001**	1.268	3.55 (0.36–34.25)	0.272
Radiotherapy	1.686	5.4 (1.82–16.02)	**0.002**	1.254	3.50 (0.90–13.60)	0.069
Nodal involvment	2.511	12.31 (4.99–10.33)	**0.00001**	1.125	3.08 (0.62–15.33)	0.168
p16 expression	0.722	2.059 (0.70–6.01)	0.186	1.126	3.08 (0.62–15.33)	0.169
HPV	0.173	1.19 (0.52–2.70)	0.678	0.915	2.49 (0.6–10.35)	0.207
*PIK3CA* mutation	0.233	1.26 (0.38–4.19)	0.704	0.394	1.48 (0.18–12.40)	0.716

**Table 4:** *PIK3CA* mutations and amplifications in HNSCC, breast cancer and PROS (Abbreviations: HNSCC—head and neck squamous cell carcinoma, OSCC—oral squamous cell carcinoma, PROS-PIK3CA—related overgrowth spectrum, HPV—human papilloma virus, *PIK3CA*—phosphatidylinositol-4,5-bisphosphate 3-kinase catalytic subunit alpha gene, FFPE—formalin-fixed paraffin-embedded, FB—fibroblast, qPCR—real time quantitative polymerase chain reaction, UDT-Seq—ultra-deep targeted sequencing, NGS—next generation sequencing, ddPCR—droplet digital PCR, BEAMing—beads, emulsification, amplification and magnetics).

Study	Year	Population	Sample Size	PIK3CA Mutations (%)	Domain	Condition	Sample	Method (%)	Associated Factors (%)		
									Alcohol	Tobacco	HPV
Sayáns et al. [23]	2019	Spain	528	amplification 21%		HNSCC	tissue		67	77	7
Schmidt et al. [51]	2018	Australia	29	E545K (31)	helical	HNSCC	Plasma	qPCR	-	-	48
Zhang et al. [46]	2016	USA	36			HNSCC	-	-	-	-	50
Feldman et al. [19]	2015	USA	421	E542K (3), E545K (5), H1047R (2%), E726K (0.2), D549H (0.2), E726K (0.2), H1048R (0.2), E545Q (0.2), Q546K (0.2), Q546P (0.2), Y1021C (0.2), R1023P (0.2), M1043I (0.2), P104L (0.2), E109del (0.2)	helical, kinase	HNSCC	FFPE	Sanger (22), NGS (78)	-	-	44
Kommineni et al. [53]	2015	India	279 (129 HNSCC and 150 controls)	E545A (47 in HNSCC and 19 in controls)	helical	HNSCC	Frozen tissue	-	-	-	-
Seiwert et al. [20]	2015	USA	120	E542K, E545K, H1047R	helical, kinase	HNSCC	tissue	NGS	59	55	42
Wirtz et al. [54]	2015	USA	22?	E545K, H1047R (14?)	helical, kinase	HNSCC	cell lines	-	-	-	-
Peng et al. [55]	2015	Taiwan	310	amplification		OSCC	FFPE	UDT-Seq	-	82%	-
Rodriguez et al. [56]	2019	Spain	29	H1047R (28), E545K (17), H1047L (10), E542K (10), Q546R (7), G1049R (3), M1043I and N1044H (3)	helical, kinase	Breast cancer	Frozen tissue	NGS	-	-	-
				H1047R (3), H1047L (3), L551I (3), E545K (3), E542K (3), G1049R (3)	helical, kinase		Plasma		-	-	-
Kodahl et al. [57]	2018	Denmark	60	E542K and E545K (13), H1047L and H1047R (27)	helical, kinase	Breast cancer	FFPE	ddPCR	-	-	-
				83% with detectable PIK3CA mutations in the tissue have similar mutations			serum				
Shimoi et al. [48]	2018	Japan	309	E542K (5), E545K (6), H1047R (23)	helical, kinase	Breast cancer	Frozen tissue, FFPE	ddPCR	-	-	-
Higgins et al. [49]	2012	USA	49	E545K; H1047R; H1047L (29 plasma and FFPE)	helical, kinase	Breast cancer	FFPE, plasma	PCR, BEAMing	-	-	-
			60	E545K; H1047R; H1047L (28 plasma and 27 FFPE)	helical, kinase						
Piacitelli et al. [50]	2018	USA	1 (27 tissues)	E545K (19)	helical	PROS	Fresh tissue	ddPCR	-	-	-
			22	E453K (9), Q546R (5), E542K (14), H1047R (5), K111_N114del (5), N1044S (5)	helical, kinase		Frozen tissue, FB, Blood, FFPE	NGS	-	-	-
